# TGR5 Signaling in Hepatic Metabolic Health

**DOI:** 10.3390/nu12092598

**Published:** 2020-08-26

**Authors:** Marlena M. Holter, Margot K. Chirikjian, Viraj N. Govani, Bethany P. Cummings

**Affiliations:** Department of Biomedical Sciences, Cornell University College of Veterinary Medicine, Ithaca, NY 14853, USA; mmh277@cornell.edu (M.M.H.); mkc224@cornell.edu (M.K.C.); vng6@cornell.edu (V.N.G.)

**Keywords:** TGR5, liver, metabolic regulation

## Abstract

TGR5 is a G protein-coupled bile acid receptor that is increasingly recognized as a key regulator of glucose homeostasis. While the role of TGR5 signaling in immune cells, adipocytes and enteroendocrine L cells in metabolic regulation has been well described and extensively reviewed, the impact of TGR5-mediated effects on hepatic physiology and pathophysiology in metabolic regulation has received less attention. Recent studies suggest that TGR5 signaling contributes to improvements in hepatic insulin signaling and decreased hepatic inflammation, as well as metabolically beneficial improvements in bile acid profile. Additionally, TGR5 signaling has been associated with reduced hepatic steatosis and liver fibrosis, and improved liver function. Despite the beneficial effects of TGR5 signaling on metabolic health, TGR5-mediated gallstone formation and gallbladder filling complicate therapeutic targeting of TGR5 signaling. To this end, there is a growing need to identify cell type-specific effects of hepatic TGR5 signaling to begin to identify and target the downstream effectors of TGR5 signaling. Herein, we describe and integrate recent advances in our understanding of the impact of TGR5 signaling on liver physiology and how its effects on the liver integrate more broadly with whole body glucose regulation.

## 1. Introduction

TGR5 is a transmembrane G-protein coupled receptor (GPCR) for bile acids that is ubiquitously expressed in mouse and human tissues [1,2,3,4,5,6]. Bile acids are amphipathic steroid molecules, synthesized from cholesterol in the liver, that signal through the nuclear receptor, Farnesoid X receptor (FXR), and TGR5, to regulate lipid, glucose, and energy metabolism and to maintain metabolic homeostasis [7,8,9]. Dysregulated bile acid signaling has been implicated in the pathogenesis of insulin resistance and type 2 diabetes [10,11,12]. Initial studies revealed that TGR5 signaling regulates glucose tolerance, inflammation, and energy expenditure [9,13,14,15], such that TGR5 is now recognized as a potential target for the treatment of metabolic disorders. To this end, much metabolic research regarding TGR5 signaling has focused on TGR5-mediated effects on GLP-1 secretion from enteroendocrine L cells [9,16,17], mitochondrial thermogenesis in adipocytes [13,18,19], and decreased inflammatory cytokine secretion from immune cells [1,14,15]. 

While the role of TGR5 signaling in immune cells, adipocytes, and enteroendocrine L cells has been well described and extensively reviewed, the impact of TGR5 on hepatic physiology and pathophysiology has received less attention. TGR5 is highly expressed in the non-parenchymal cell-types of the liver [20,21,22,23] and has been recently identified in hepatocytes [24]. Examination of the role of TGR5 signaling in these various cell types has contributed to a growing body of literature highlighting TGR5 as a key contributor to liver physiology and pathophysiology. Genetic ablation of TGR5 has been shown to make mice more susceptible to impaired glucose tolerance, hepatic insulin resistance, hepatic steatosis and fibrosis, as well as altered bile acid metabolism [9,25,26]. Additionally, treatment of mice with TGR5-specific agonists has been shown to ameliorate many of these metabolic impairments, underscoring the potential therapeutic nature of TGR5 in treating metabolic disease [7,9,13,24,27,28,29,30]. However, the clinical development of TGR5 agonists is complicated by the breadth of effects associated with systemic TGR5 activation, particularly in regard to TGR5-specific effects on gallbladder filling and gallstone formation [22,24,30,31]. This review will summarize the currently available knowledge on the impact of TGR5 on hepatic physiology, its influences on metabolic regulation, and the knowledge gaps that need to be addressed to enable effective therapeutic targeting of the TGR5 signaling for the treatment of metabolic disease. 

## 2. Hepatic TGR5 Signaling and Expression 

TGR5 is a member of the rhodopsin-like family of GPCRs [2]. TGR5 is encoded by a single-exon gene and is widely conserved among vertebrates [2]. TGR5 is activated by several bile acids, with lithocholic acid (LCA) as the most potent natural agonist, followed by deoxycholic acid (DCA), chenodeoxycholic acid (CDCA) and cholic acid (CA) [2]. Further, taurine conjugation of bile acids increases the affinity for TGR5, as compared to unconjugated bile acids; however, conjugation with glycine has a negligible impact on TGR5 affinity [32]. Based on the robust metabolic effects of TGR5 signaling, multiple synthetic [3,9,13,30,33,34,35] and semi-synthetic [28] TGR5-specific agonists have been designed and synthesized, in addition to other natural ligands that have been identified [16,29]. 

In 2002, Maryuama et al., discovered TGR5 as a novel GPCR that, in response to bile acids, enhanced cAMP production in human cell lines [2]. Similarly, Kawamata et al., reported that treatment of a TGR5-expressing human monocytic cell line (THP-1) with taurolithocholic acid (TLCA), LCA, and DCA, dramatically increased cAMP production and suppressed cytokine production [1]. Together, these studies identified that, in most cell types, TGR5 is coupled to a stimulatory G-alpha-protein (Gα_s_), whereby ligand binding to TGR5 results in the activation of adenylyl cyclase-cAMP-PKA signaling and the associated downstream effects, including the recruitment of cAMP response element-binding protein (CREB) to target genes [1,2]. However, further characterization of TGR5 expression and function in other cell types has demonstrated that TGR5 signaling activates multiple different kinase pathways in addition to PKA, including protein kinase B (AKT) [3], Rho kinase [4], mammalian target of rapamycin complex 1 (mTORC1) [3], as well as extracellular signal-related kinase 1/2 (ERK1/2) [5,6]. 

TGR5 is ubiquitously expressed in rodent and human tissues and has a well-defined role in many regulatory functions that affect whole body metabolism [9,13,15,20]. TGR5 is expressed in endocrine glands, the kidney, adipocytes, muscle, the central nervous system, immune cells, spleen, lung, the gastrointestinal tract (mainly in the ileum and colon), and the enteric nervous system [1,2,36]. TGR5 is also highly expressed in the liver, specifically within non-parenchymal cells, including Kupffer cells [20], sinusoidal endothelial cells [21], and cholangiocytes [22,23]. While it has been speculated that TGR5 is not expressed in hepatocytes, we are not aware of any previously published data demonstrating this. Recently, we reported that TGR5 is expressed in mouse hepatocytes [24], which is in line with previous work in which TGR5 expression was detected in a human hepatocellular carcinoma cell line [37] and in canine hepatocytes [38]. 

**Figure 1:**
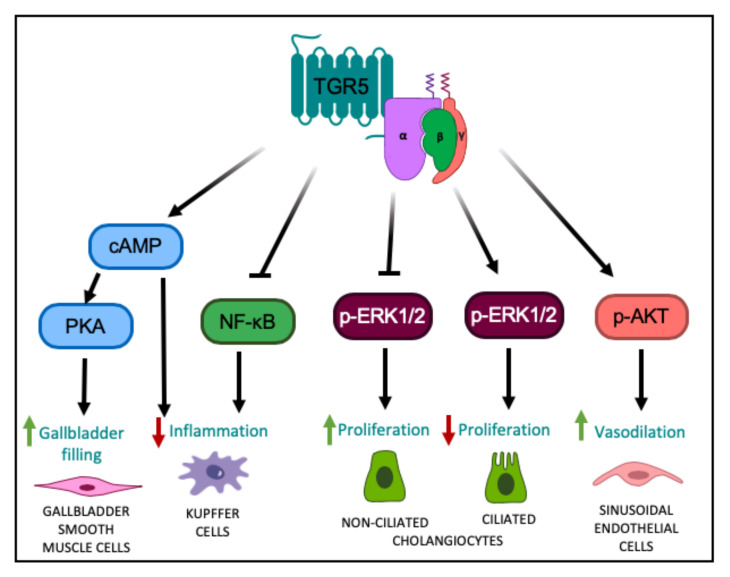
TGR5-mediated cell signaling pathways in different liver cell types. TGR5 activation leads to increased intracellular cAMP levels, followed by activation of PKA, ultimately leading to altered gene expression [1,2], resulting in relaxation of gallbladder smooth muscle cells and increased gallbladder filling. TGR5 signaling in Kupffer cells decreases LPS-induced cytokine production via a cAMP-dependent pathway [20] and antagonizes NF-κB, both resulting in a decreased hepatic inflammatory response [15,39]. TGR5 signaling in non-ciliated cholangiocytes inhibits ERK1/2 signaling, resulting in increased proliferation; whereas in ciliated cholangiocytes, TGR5 signaling increases ERK1/2 activity to decrease proliferation [5]. TGR5 signaling in liver sinusoidal endothelial cells enhances AKT phosphorylation and increases vasodilation [45,46].

A growing body of literature suggests that TGR5 signaling plays an essential role in hepatic metabolic regulation. Our group recently identified a previously unknown role for hepatocyte TGR5 signaling in regulating whole body glucose homeostasis and insulin sensitivity; however, the underlying signaling pathway through which this occurs remains unresolved [24]. Moreover, other studies have characterized anti-inflammatory, choleretic, proliferative, and protective effects associated with TGR5 signaling in the nonparenchymal cell types of the liver. For instance, in Kupffer cells and resident macrophages, TGR5 activation dampens the hepatic inflammatory response through the attenuation of LPS-induced cytokine production, via the classical TGR5-cAMP-dependent pathway [20], and also through a reduction in NF-κB-dependent inflammatory responses [15] (Figure 1). TGR5 signaling antagonizes NF-κB by decreasing the phosphorylation of IκBα, the nuclear translocation of p65, and NF-κB DNA binding activity [15,39]. Through these separate pathways, TGR5 signaling functions to diminish hepatic inflammation. 

The function of TGR5 signaling within the biliary tree has also been rigorously studied. Ligand binding to TGR5 in cholangiocytes induces CFTR-dependent chloride and bicarbonate secretion into bile, which enhances choleresis and forms a bicarbonate umbrella to protect the liver parenchyma from bile acid toxicity [23,40,41,42]. TGR5 signaling in cholangiocytes has also been shown to inhibit, as well as promote, cell proliferation depending on the subcellular localization of the TGR5 receptor. In non-ciliated cholangiocytes where TGR5 is localized to the apical membrane, TGR5 couples to Gα_s_ to increase intracellular cAMP levels, resulting in increased ERK1/2 signaling and promotion of proliferation (Figure 1) [5]. In contrast, in ciliated cholangiocytes where TGR5 is localized to the cilia, TGR5 couples to Gα_i_ to decrease intracellular cAMP levels, resulting in increased ERK1/2 signaling and inhibition of proliferation (Figure 1) [5]. TGR5 is also expressed in the smooth muscle cells of the gallbladder [43]. TGR5 signaling in these cells activates the cAMP-PKA pathway and causes hyperpolarization of smooth muscle cells by opening the K_ATP_ channels, ultimately leading to inhibition of gallbladder contractility and increased gallbladder filling [43] (Figure 1). Through these combined functions, TGR5 signaling protects the liver and biliary tree from the cytotoxic effects of bile acids. This is further underscored by the finding that whole body *Tgr5^−/−^* mice exhibit more severe liver damage, biliary injury, and impaired cholangiocyte proliferation in response to bile acid feeding, compared to wild-type mice [6,44]. 

Furthermore, in liver sinusoidal endothelial cells, TGR5 signaling increases the generation of vasodilatory molecules, nitrogen oxide and hydrogen sulfide. More specifically, TGR5 activation results in PKA-mediated phosphorylation of endothelial nitric oxide synthase leading to increased production of nitric oxide [21]. Additionally, ligand binding to TGR5 has been shown to activate AKT in these cells, resulting in serine phosphorylation of cystathionine γ-lyase and a subsequent increase in hydrogen sulfide production [45,46]. TGR5 signaling has also been shown to inhibit expression and secretion of endothelin-1, a potent vasoconstrictor [47]. Through TGR5 signaling, the generation of vasodilatory and inhibition of vasoconstrictor molecules serves to modulate liver microcirculation, mitigate portal hypertension and enable adaptation of hepatic blood flow to nutrient uptake [45,47].

## 3. TGR5 Regulation of Liver Health and Its Impact on Metabolic Disease

### 3.1. TGR5 and Glucose Regulation

**Figure 2:**
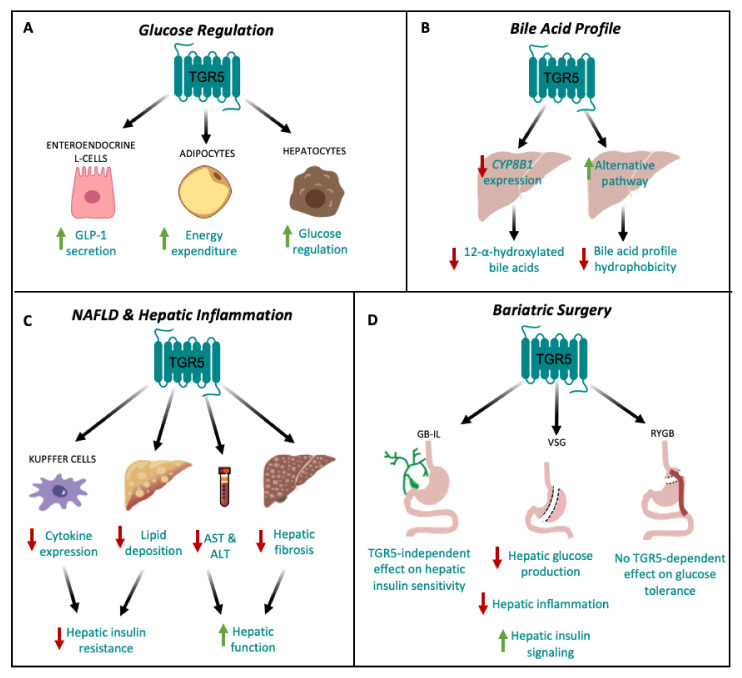
TGR5 signaling in hepatic metabolic regulation. (**A**) TGR5 signaling regulates glucose homeostasis by increasing GLP-1 secretion from enteroendocrine L cells [9,16] and energy expenditure in adipocytes [13]. TGR5 signaling also improves glucose tolerance through signaling in hepatocytes [24]. (**B**) TGR5 signaling decreases hepatic CYP8B1 expression, leading to a decrease in the production of 12-α-hydroxylated bile acids [26,33], and upregulates the alternative pathway of bile acid synthesis, thereby decreasing bile acid profile hydrophobicity [7,26]. (**C**) TGR5 signaling may protect against non-alcoholic fatty liver disease (NAFLD) by reducing hepatic cytokine expression in Kupffer cells [15,20] and decreasing hepatic lipid deposition [9,27], which together, attenuate hepatic insulin resistance. TGR5 signaling also decreases serum AST and ALT concentrations [9], as well as hepatic fibrosis [9], ultimately improving hepatic function. (**D**) TGR5-dependent improvements in glucose tolerance following bariatric surgery depend on surgery type. Vertical sleeve gastrectomy (VSG) results in decreased hepatic glucose production and inflammation, and increased hepatic insulin signaling [33,74]. Following gallbladder bile diversion to the ileum (GB-IL), there is no TGR5-dependent improvement in insulin sensitivity [76]. Roux-en-Y gastric bypass (RYGB) has no TGR5-dependent effect on glucose tolerance [77].

Targeted disruption of TGR5 in mouse models of metabolic disease has identified a role for TGR5 signaling in the maintenance of whole body glucose homeostasis and insulin sensitivity. For instance, Thomas et al., have shown that mice with a gain-of-function of TGR5 have improved glucose tolerance, whereas whole body *Tgr5^−/−^* mice have impaired glucose tolerance during an oral glucose tolerance test as compared to weight-matched, high fat diet (HFD)-fed controls [9]. Previous work has also shown that whole body *Tgr5^−/−^* mice fed a HFD are more insulin-resistant than *Tgr5^+/+^* mice, as assessed by an insulin tolerance test [25]. Furthermore, TGR5 agonists have been shown to ameliorate glucose intolerance and improve insulin resistance in obese and diabetic mice [9,13,27,28,29,48]. Treatment of diet-induced obese (DIO) mice with INT-777, a semi-synthetic TGR5 agonist, has been shown to promote GLP-1 secretion from L-cells, improve insulin sensitivity in liver and muscle and decrease hepatic glucose production [9]. Compound 18, a potent and highly-specific TGR5 agonist, has been shown to robustly induce GLP-1 secretion and improve glucose tolerance in HFD-fed *Tgr5^+/+^* mice, but not in whole body *Tgr5^−/−^* mice [30]. Similarly, chronic treatment with oleanolic acid, an extract from *Olea europaea* and a TGR5 agonist, decreased fasting plasma insulin concentrations and improved glucose tolerance in mice [29]. These results also have translational relevance in humans, as a trend towards reduced hyperglycemia and elevated GLP-1 secretion was also seen in a group of patients with type 2 diabetes who received a high dose of the TGR5 agonist, SB-756050 [35]. Additionally, Potthoff et al., reported that treatment of DIO mice with the bile acid sequestrant, colesevelam, significantly reduced hyperinsulinemia and improved glucose tolerance through a decrease in hepatic glucose production, an effect that was absent in whole body *Tgr5^−/−^* mice [49]. Until recently, the effect of TGR5 signaling to improve glucose tolerance and insulin sensitivity has been attributed primarily to its effects on mitochondrial function in adipose tissue and GLP-1 secretion from enteroendocrine L cells, leading to insulin release from the pancreas (Figure 2A) [9,13,16,17,34,50]. 

However, since the liver is central to whole body glucose regulation, this suggests that hepatic TGR5 signaling may be a critical contributor to the overall metabolic benefits of TGR5. Therefore, we administered Compound 18 to hepatocyte-specific TGR5 knockout mice to assess the role of hepatocyte TGR5 on glucose regulation [30]. We found that administration of Compound 18 improves glucose tolerance and insulin sensitivity in HFD-fed *Tgr5^HEP+/+^* mice, but not in *Tgr5^Hep−/−^* mice. Furthermore, we found that this effect occurred independent of body weight and GLP-1 secretion [24]. Therefore, our findings demonstrate that TGR5 agonists improve glucose homeostasis through an additional, novel mechanism specific to hepatocyte TGR5 signaling (Figure 2A). However, the mechanisms through which hepatocyte TGR5 signaling improves glucose tolerance remain undefined. Further understanding of how liver TGR5 regulates glucose homeostasis is warranted but will necessitate further cell-type specific in vivo studies of hepatic TGR5 function.

### 3.2. TGR5 and Hepatic Bile Acid Metabolism

Alterations in bile acid homeostasis have been linked to the pathogenesis of insulin resistance and obesity [51,52,53,54,55]. Increased bile acid profile hydrophobicity and increased 12-α-hydroxylated bile acids [56], are associated with insulin resistance and type 2 diabetes in humans [57]. Conversely, administration of hydrophilic bile acids, such as ursodeoxycholic acid (UDCA), has been shown to improve insulin sensitivity in mouse models and patients with type 2 diabetes [58,59,60]. Therefore, understanding bile acid profile regulation may enable targeting of endogenous bile acid profile for the treatment of metabolic disease. To this end, a growing body of literature has highlighted the role of TGR5 in regulating bile acid profile. Mice with TGR5 ablation have a decreased total bile acid pool size [2,22,31], increased 12-α-hydroxylated bile acids and increased hydrophobic bile acids [26,31]. For example, Pean et al., found that whole body *Tgr5^−/−^* mice exhibited more hydrophobic biliary, circulating, and hepatic bile acid pool compositions, as well as decreased muricholic acid (MCA)/CA ratios in both the plasma and liver, as compared to wild-type controls [44]. Furthermore, Donepudi et al., previously found that whole body *Tgr5^−/−^* mice had an increased ratio of 12-α-hydroxylated bile acids to non-12-α-hydroxylated bile acids, which was specifically characterized by increased taurocholic acid (TCA) and decreased tauromuricholic acid (TMCA) [26]. Similarly, we reported that TGR5 signaling decreases circulating bile acid profile hydrophobicity following vertical sleeve gastrectomy (VSG) in mice, which was associated with a TGR5-dependent improvement in glucose tolerance [33]. The results of these studies suggest that TGR5 may play an important role in altering bile acid composition, specifically through a reduction in 12-α-hydroxylated bile acids. However, as bile acids are known to regulate lipid, glucose and energy homeostasis through activation of both FXR and TGR5, Pathak et al., administered INT-767, a dual FXR and TGR5 agonist, OCA, an FXR agonist, and INT-777, a TGR5 agonist, to HFD-fed mice to assess the effects of FXR and TGR5 signaling, alone and in combination, on hepatic bile acid metabolism [7]. INT-767 decreased TCA and CA, but increased TMCA, resulting in a decrease in hydrophobicity of the gallbladder bile acid pool [7]. Furthermore, INT-767 was most effective at improving hepatic insulin signaling and hepatic bile acid, lipid, and glucose metabolism [7]. Overall, these studies suggest that decreasing bile acid pool hydrophobicity through increased TGR5 signaling has the potential to improve hepatic glucose metabolism and hepatic insulin signaling; and further, that the most therapeutically efficacious way to do so may be the combinatorial targeting of TGR5 and FXR.

However, the mechanisms by which TGR5 regulates bile acid pool composition are not completely understood. Several studies have identified a role for TGR5 in modulating expression of the enzymes that regulate bile acid synthesis in the liver. CYP7A1 is the rate-limiting enzyme for hepatic bile acid synthesis and CYP8B1 is the hepatic enzyme required for the synthesis of 12-α-hydroxylated bile acids. Donepudi et al., reported that hepatic *Cyp7a1* expression was similar between whole body *Tgr5^+/+^* and *Tgr5^−/−^* mice, but that fasting induction of hepatic *Cyp8b1* was attenuated in whole body *Tgr5^−/−^* mice [26]. Similarly, we previously reported that the decrease in circulating bile acid profile hydrophobicity following VSG in mice was associated with a TGR5-dependent reduction in hepatic CYP8B1 protein expression, with no effect on CYP7A1 expression [33]. In line with this, Pathak et al., found that treatment of mice with OCA (an FXR-specific agonist) or INT-767 resulted in decreased hepatic *Cyp7a1* and *Cyp8b1* expression, whereas treatment with INT-777 only decreased hepatic *Cyp8b1* expression [7]. Together, these studies suggest that one mechanism through which TGR5 may regulate bile acid profile is through the selective downregulation of hepatic *Cyp8b1* expression (Figure 2B). Moreover, Donepudi et al., also reported a decrease in liver *Cyp7b1* and *Cyp27a1* levels, enzymes involved in the alternative pathway of bile acid synthesis, in whole body *Tgr5^−/−^* mice [26]. Similarly, Pathak et al., reported that treatment of DIO mice with INT-767 stimulates expression of *Cyp7b1* and *Cyp27a1* [7]. These findings suggest that TGR5 signaling may also upregulate the alternative pathway of bile acid synthesis, which reduces TCA and CA and increases TMCA, thereby decreasing the hydrophobicity of the bile acid pool (Figure 2B).

Another potential mechanism whereby TGR5 signaling regulates bile acid profile is through the selective reabsorption of hydrophobic bile acids through the biliary epithelium, a process called cholehepatic shunting. This hypothesis first gained traction when TGR5 was detected in colocalization with the apical sodium-dependent bile acid transporter (ABST) in gallbladder epithelial cells. Upon activation of TGR5, the subsequent increase in cAMP resulted in insertion of ABST into the apical membrane, leading to enhanced uptake of bile acids from bile into biliary epithelial cells [23]. Through this direct reabsorption into biliary epithelial cells, this shunt is hypothesized to restrict the hydrophobicity of the bile acid pool through the selective reabsorption of secondary bile acids [61]. To this end, Jourdainne et al., reported that through increased gallbladder dilation, TGR5 signaling may favor cholehepatic shunting, thereby increasing the ratio of primary to secondary bile acids [62]. However, the precise signaling and molecular mechanism operating this shunt remain to be explored. Overall, these studies provide ample evidence for a role of TGR5 signaling in the regulation of bile acid profile; however, the mechanisms through which this occurs remain incompletely understood and requires further study in tissue-specific knockout mouse models.

### 3.3. TGR5 and Hepatic Inflammation

Chronic inflammation is increasingly recognized as a key driver of insulin resistance [63]. Metabolic diseases are often characterized by abnormal cytokine production, increased acute-phase reactants, and activation of a network of inflammatory signaling pathways. Of note, the architectural organization of the liver is such that the metabolic cells, hepatocytes, are in close proximity to the immune cells, the Kupffer cells. This organization enables continuous and dynamic interactions between the metabolic and immune cells of the liver. Thus, the chronic inflammatory signaling associated with obesity promotes hepatic insulin resistance [64]. Moreover, under healthy conditions, hepatic insulin receptor signaling downregulates expression of key gluconeogenic enzymes. Therefore, in the presence of hepatic insulin resistance, endogenous hepatic glucose production is elevated, which contributes to whole body glucose dysregulation. As a therapeutic drug target for treating metabolic disease, TGR5 has been shown to have anti-inflammatory properties in the liver. For example, in response to liver injury, whole body *Tgr5^−/−^* mice experience exacerbated inflammatory responses and hepatic fibrosis compared to wild-type controls [65]. In line with this, various in vitro and in vivo studies have highlighted the role of TGR5 in the suppression of macrophage and Kupffer cell functions in response to bile acid treatment or stimulation by TGR5 agonists [1,15,20]. More specifically, in Kupffer cells and macrophages, TGR5 signaling leads to activation of a cAMP-dependent pathway that decreases LPS-induced cytokine expression and the NF-κB-dependent inflammatory response, thereby reducing hepatic inflammation [15,20]. This is particularly relevant to the therapeutic potential of TGR5 agonists as inhibition of NF-κB related inflammation has been shown to improve glucose tolerance in vivo [66]. Together, these studies suggest that the anti-inflammatory properties of hepatic TGR5 signaling may protect against the development and progression of hepatic insulin resistance to ultimately improve whole body glucose regulation (Figure 2C).

### 3.4. TGR5 and Bariatric Surgery

Bariatric surgery is currently the most effective treatment for obesity and is associated with high rates of T2DM remission [67,68]. Many of the early metabolic benefits of bariatric surgery, including improved insulin and glucose handling, occur prior to significant weight loss [67], which suggests that other hormonal or metabolic mediators may be driving these effects. Bariatric surgery has been shown to increase circulating bile acid concentrations in both humans and rodent models [33,69,70], which has led to the hypothesis that enhanced bile acid signaling may underlie the metabolic benefits of bariatric surgery. The role of bile acid signaling in bariatric surgery has been previously reviewed [71,72,73]. Here, however, we have focused on the role TGR5 in the effects of bariatric surgery on hepatic metabolism. Various studies have reported TGR5-dependent improvements in glucose tolerance following bariatric surgery, which was associated with improved hepatic insulin signaling (Figure 2D) [33,74]. For example, Ding et al., reported a TGR5-dependent improvement in whole body insulin sensitivity, as assessed by hyperinsulinemic-euglycemic clamp, as well as markedly suppressed hepatic glucose production following VSG in mice [74]. These results suggest that suppression of hepatic glucose production and elevation of peripheral glucose utilization both contributed to improved insulin sensitivity in their model. Similarly, our lab reported a TGR5-dependent improvement in hepatic insulin signaling following VSG in mice; and further, that this effect was associated with reduced hepatic inflammatory cytokine expression, suggesting that reduced inflammation improved hepatic insulin signaling to improve glucose tolerance [33]. Additionally, similar to the effect of TGR5 on the beneficial outcomes of VSG, Ryan et al., reported that FXR contributes to improvements in weight loss and glycemic control following VSG in mice [75], which provides further support for a combinatorial role of TGR5 and FXR in regulating whole body metabolism. However, additional studies in tissue-specific mouse models are needed to identify the cell-type(s) driving these metabolic benefits. Comparatively, Albaugh et al., detected a TGR5-independent improvement in glucose tolerance following bile diversion to the ileum (GB-IL) in mice, in which improvements in glucose tolerance following GB-IL was primarily due to a TGR5-independent effect of bile acids on improved hepatic insulin sensitivity [76]. Conversely, the results on the role of TGR5 in the metabolic benefits of Roux-en-Y gastric bypass (RYGB) have been mixed [77,78]. Zhai et al., suggest that ileal deoxycholic acid-TGR5-mTORC1 signaling contributes to increased GLP-1 production following RYGB in mice [78]. In contrast, Hao et al., reported similar improvements in glucose tolerance and insulin resistance, as assessed by insulin tolerance testing, in whole body *Tgr5^+/+^* and *Tgr5^−/−^* mice following RYGB [77]. Overall, the differential effects of whole body TGR5 signaling on hepatic metabolic outcomes following bariatric surgery could stem from differences in the underlying mechanisms through which surgery types improve metabolic regulation. Furthermore, this points to VSG as a particularly effective surgical model for the assessment of how liver TGR5 signaling contributes to whole body metabolic regulation (Figure 2D).

## 4. TGR5 and Hepatic Lipid Metabolism

Non-alcoholic fatty liver disease (NAFLD) is a common co-morbidity of obesity and develops due to an accumulation of lipid in the liver. NAFLD can progress to non-alcoholic steatohepatitis (NASH), characterized by inflammation, progressive fibrosis, and hepatocellular damage and can eventually progress to cirrhosis or hepatobiliary cancer. Loss of TGR5 signaling has been shown to promote liver lipid deposition in preclinical studies, suggesting that TGR5 signaling may protect against NAFLD. For example, HFD-fed male whole body *Tgr5^−/−^* mice exhibit increased liver lipid deposition compared with male wild-type mice. Furthermore, HFD-fed male whole body *Tgr5^−/−^* mice exhibit increased liver lipid deposition compared with female whole body *Tgr5^−/−^* mice, suggesting that the effect of TGR5 signaling on liver lipid deposition may be influenced by sex [25].

Studies investigating the effects of TGR5 agonists on NAFLD further demonstrate a role for TGR5 in protection against liver lipid deposition. Treatment of HFD-fed mice with INT-777 decreased plasma free fatty acids and liver steatosis, liver fibrosis and plasma AST and ALT levels [9]. Similarly, the TGR5 agonist, RDX8940, was shown to decrease liver weight and hepatic triglyceride and cholesterol levels in mice fed a Western diet [27]. Moreover, dual agonists of TGR5 and FXR protect against NAFLD. For example, treatment of DIO mice with INT-767 decreased hepatic and serum triglycerides and cholesterol levels in mice [7]. Similarly, long-term administration of INT-767 decreased serum AST and ALT concentrations, as well as decreased hypoxia, lipid accumulation, collagen deposition and mononuclear cell infiltrates in the livers of a rabbit model of HFD-induced metabolic syndrome [79]. Furthermore, INT-767 treatment reduced expression of liver pro-inflammatory genes, as well as genes related to *de novo* lipogenesis, and promoted expression of anti-inflammatory genes and genes related to lipid uptake [79]. In line with this, *ob/ob* mice on a HFD supplemented with trans-fat, cholesterol, and fructose treated with INT-767 exhibited a greater decrease in liver parenchymal fibrosis and inflammatory infiltrates compared to mice treated with OCA [80]. Moreover, treatment of mice on HFD supplemented with fructose with BAR502, another dual FXR and TGR5 agonist, reduced hepatic steatosis, inflammation and fibrosis [81]. Although we cannot discern the contribution of TGR5 to the beneficial metabolic effects of dual TGR5/FXR agonists on hepatic function, these findings suggest that the combinatorial targeting of FXR and TGR5 is a promising therapeutic modality for improving hepatic function.

Studies in mouse models of bariatric surgery report conflicting results on the role of TGR5 in the effect of bariatric surgery to reduce liver lipid deposition. Ding et al., reported that VSG decreased hepatic steatosis in wild-type but not in whole body *Tgr5^−/−^* mice [74]. In contrast, our lab reported TGR5-independent improvements in hepatic triglyceride content following VSG in mice [33]. The discrepancy between these findings could be explained by differences in the whole body *Tgr5^−/−^* mouse models used, duration of HFD-feeding, and variation in diet composition in each study [74].

Overall, these findings suggest that enhanced TGR5 signaling attenuates hepatic triglyceride accumulation and fibrosis, and improves liver function; however, the mechanisms through which this occurs remain unknown (Figure 2C). Further work is needed to address this limitation to more completely harness the therapeutic potential of TGR5 in patients with NAFLD. Moreover, these studies suggest that, similar to the regulation of bile acid profile, the combinatorial targeting of TGR5 and FXR may provide the most therapeutic utility.

## 5. Negative Side-Effects of Enhanced Hepatic TGR5 Signaling

**Figure 3:**
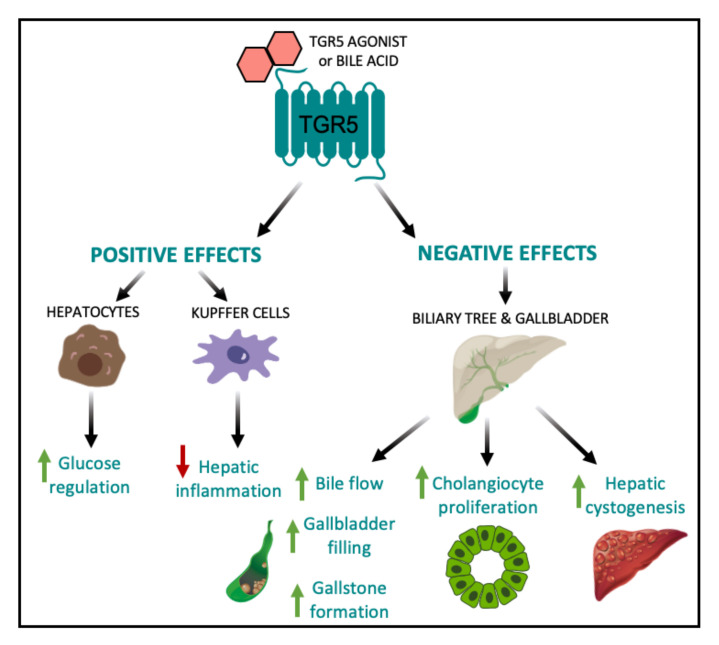
Positive and negative effects of elevated hepatic TGR5 signaling. Within the liver, TGR5 activation by TGR5-specific agonists and/or bile acids leads to beneficial metabolic outcomes, including improved glucose tolerance [24] and decreased hepatic inflammation [15,20]. However, TGR5 signaling in the biliary tract and gallbladder results in increased bile flow, gallbladder filling, and gallstone formation [22,24,28,29,31,82,83], as well as increased cholangiocyte proliferation and hepatic cystogenesis [84].

Therapeutic targeting of TGR5 for the treatment of metabolic disease has been hindered by the wide range of effects associated with systemic TGR5 activation. In particular, a major side effect of TGR5 agonists is the inhibition of gallbladder emptying, leading to gallstone formation and cholestasis. TGR5 is highly expressed in the gallbladder and cholangiocytes [23]. TGR5 signaling stimulates relaxation of gallbladder smooth muscle cells to induce gallbladder filling [43]. Several studies have reported that the oral administration of the TGR5 agonists, such as INT-777, LCA, oleanolic acid, Compound 23g, Compound 18, and RO552739, increase biliary bile flow and gallbladder filling in wild type mice [24,28,29,31,82,83]. In line with this, several studies have reported reduced gallbladder volumes and reduced bile flow in whole body *Tgr5^−/−^* mice relative to wild-type controls [31,43]. However, whole body *Tgr5^−/−^* mice are protected against the formation of cholesterol crystals and gallstones when fed a lithogenic diet [22], which suggests that the continual build-up of bile in the biliary tract has the potential to cause cholestasis and indirect liver injury. Therefore, despite the robust metabolic benefits associated with hepatic TGR5 signaling, chronic stimulation of TGR5 within the biliary tract complicates the task of developing a safe and efficacious TGR5 agonist (Figure 3).

TGR5 expression in the biliary tract is also crucial for protection against bile acid overload. For example, TGR5 signaling strengthens the biliary epithelial barrier resulting in decreased hepatocyte necrosis in the presence of obstructive cholestasis in mice [42]. In contrast to this protective role, TGR5 activation in the biliary tract has been shown to contribute to the development of polycystic liver disease and cholangiocarcinoma [6,84]. Whole body *Tgr5^−/−^* mice exhibit decreased cholangiocyte and hepatocyte proliferation and increased liver injury in response to cholestatic stress [6]. In addition, taurolithocholic acid and oleanolic acid stimulation of TGR5 in vitro and in vivo have been shown to promote cholangiocyte proliferation through TGR5-dependent increases in cAMP production. However, this increased proliferation was accompanied by increased hepatic cystogenesis and the development of polycystic liver disease (Figure 3) [84]. Comparatively, treatment of cultured cystic cholangiocytes with the TGR5 antagonist, SBI-115, decreased proliferation, cholangiocyte spheroid growth and cAMP levels [84], suggesting that TGR5 inhibition may be a promising therapeutic approach to polycystic liver disease and treating malignant cholangiocytes. Therefore, while the proliferative and anti-apoptotic properties of TGR5 are crucial for hepatoprotection under conditions of bile acid toxicity, it also impedes the regulation of malignant cholangiocytes, which may lead to an increased risk of developing cholangiocarcinoma and liver cancer [6]. To this end, the negative effects of TGR5 agonists on cholestasis, cholangiocyte proliferation, and hepatic cystogenesis decreases the enthusiasm of therapeutically targeting TGR5 to treat liver disease (Figure 3).

## 6. Conclusions

In light of the ubiquitous expression of TGR5 throughout the liver, hepatic TGR5 signaling is increasingly recognized as a key contributor to whole body metabolic regulation. Nevertheless, the wide range of effects associated with liver TGR5 signaling complicates therapeutic targeting of TGR5. Further delineation of the downstream mediators of TGR5 signaling in the liver is needed to enable rational development of compounds targeting mediators of the metabolically beneficial actions of TGR5 in the liver. Such a compound could be coupled with an GLP-1 receptor agonist to develop a highly effective multimodal therapy for the treatment of type 2 diabetes.
